# Phytochemical Investigation of Ginger: Unveiling Therapeutic Compounds With Multiple Health Applications

**DOI:** 10.1155/ijfo/4311760

**Published:** 2025-10-28

**Authors:** Bazghah Sajjad, Arusa Aftab, Zubaida Yousaf, Zainab Maqbool, Wei Sun, Humaira Rizwana

**Affiliations:** ^1^ Department of Botany, Lahore College for Women University, Lahore, Pakistan, lcwu.edu.pk; ^2^ Institute of Chinese Materia Medica, China Academy of Chinese Medical Sciences, Beijing, China, cacms.ac.cn; ^3^ Department of Botany and Microbiology, King Saud University, Riyadh, Saudi Arabia, ksu.edu.sa

**Keywords:** compounds, food, health, metabolites, pharmacology

## Abstract

Ginger, a vital food component, has a long history of usage in various forms of traditional and alternative medicine with numerous health benefits. Ginger (*Zingiber officinale* Roscoe) is considered herbal medicine, being a safer alternative with manifold active and harmless ingredients. The primary aim of this research was to conduct a phytotherapeutic profiling and potential evaluation of essential oil and aqueous extracts derived from ginger rhizomes. The rhizomes were acquired from a local market in Lahore, Punjab, Pakistan. Essential oil extraction was performed using a hydrodistillation method, yielding conc. from 200 to 3.125 mg/mL. Results indicated that both the essential oil and aqueous extracts possessed substantial total phenolic content. Furthermore, DPPH radical scavenging activity was notable at 200 mg/mL, with the essential oil showing 80.54*%* ± 1.36*%* and the aqueous extracts at 90.06*%* ± 0.85*%*. The extracts also demonstrated potent antibacterial activity against *Azospirillum lipoferum* and antifungal activity against *Trichoderma hamatum* (24 ± 2) at 200 mg mL^−1^ in comparison to standard antibiotics. Moreover, the essential oil extracts exhibited promising antidiabetic and anti‐inflammatory (83.33 ± 0.90) properties compared to commercial drugs. Gas chromatography–mass spectrometry (GCMS) and liquid chromatography–mass spectrometry (LCMS) analyses revealed the presence of multiple phytotherapeutic compounds, viz, trihexyl(tetradecyl)phosphonium bis[(trifluoromethyl)sulfonyl]imide, heptatetracontylcyclohexane, 3,5‐dihydroxybenzyl alcohol, tris(heptafluorobutyrate), tetratriacontane, 17‐hexadecyl‐, and 1‐deoxy‐1‐(methylamino)‐D‐galactitol, N,O,O,O,O,O‐hexa(trifluoroacetyl)‐, which have not been reported in *Z. officinale* previously. Thus, the plant is a highly valuable resource for the pharmaceutical industry as a safer alternative to conventional drugs.

## 1. Introduction

Plants have been used in traditional medicine for centuries and continue to be a significant source of medication for various serious diseases worldwide [1, 2]. A diverse array of phytochemicals derived from medicinal plants serves as phytotherapeutic agents [3]. Medicinal plants are a cornerstone of traditional and modern healthcare, offering a vast collection of phytotherapeutic properties derived from their unique chemical compositions. These properties refer to the beneficial effects that plants and their extracts exert on the human body, contributing to disease prevention, treatment, and overall well‐being. They also play a crucial role in the discovery of novel drugs because the pharmacological potential of plants is attributed to secondary metabolites like tannins, alkaloids, glycosides, and terpenoids. Zingiberaceae is the largest family of monocotyledons consisting of 53 genera and over 1300 species. It is commonly known as the ginger family and is remarkably an important group of plants from a phytotherapeutic perspective [4]. Many of its members, particularly those with aromatic rhizomes, have been used for centuries in traditional medicine systems like Ayurveda, traditional Chinese medicine (TCM), and various folk medicine practices worldwide and have been utilized as traditional therapy for multiple ailments [5]. The members of this family are grown in a moist and shaded environment [6]. *Zingiber officinale* Roscoe belongs to the family Zingiberaceae, originated in South East Asia, and is now grown in many regions of the world. It has been cultivated for thousands of years as a spice and for therapeutic or restorative purposes. Ginger has been used in traditional medicines to treat a number of illnesses, including rheumatism, gastrointestinal issues, sneezing, fever, influenza, vomiting, and travel sickness [7]. The ginger market is expected to reach $7.50 billion by 2033 from $4.41 billion in 2024, with a CAGR of 6.07% from 2025 to 2033, increasing government initiatives, growing advancements in extracts, and the expanding usage of ginger in organic food and pharmaceuticals (Ginger Global Strategic Report 2024) (https://www.researchandmarkets.com/reports/5923615/ginger-market-size-forecast-industry). Despite the extensive research, the experimental validations of the ginger variety have not been fully utilized in Pakistan. However, local populations in Pakistan traditionally use ginger rhizome to alleviate sore throat, rheumatism, stomachache, sprains, and physical pain. Studies support the traditional knowledge that ginger contains phytochemicals with antioxidant, antimicrobial, antidiabetic, and anti‐inflammatory properties.

Liquid chromatography–mass spectrometry (LCMS) is a highly effective technique for metabolite testing, offering significant advantages for compound detection, sensitivity, and selectivity. Its applications in metabolomics, drug metabolism, and other fields have made it an indispensable tool for understanding biological processes utilized for lipidomic analyses, involving the isolation of the hydrocarbons from the biomolecules followed by LC partitioning. The separated compounds are then ionized before being detected by a mass spectrometer for specific ions. LCMS can be performed through class‐specific methods (product‐ion scanning, precursor‐ion scanning, or neutral‐loss scanning), untargeted approaches (full spectra acquisition), or targeted techniques (multiple‐reaction monitoring) [8]. Fourier transform infrared (FTIR) is very useful in providing metabolic fingerprinting to gain the entire metabolic profile of a sample, and changes to that profile can be detected. It is a vital technique for medical diagnostics and food science [9]. Gas chromatography–mass spectrometry (GCMS) is a powerful and versatile tool for metabolite testing, offering high separation efficiency, sensitive detection, and structural identification capabilities [9]. In the present study, FTIR spectroscopy, GCMS, and LCMS were used for chemical profiling.

The present study was aimed at investigating the phytotherapeutic potential through antimicrobial, antioxidant, antidiabetic, and anti‐inflammatory effects of the essential oil (EO) and aqueous extracts of *Z. officinale* at variable concentrations.

## 2. Methods

### 2.1. Plant Material Collection and Extraction

Ginger rhizomes were purchased from the local market of dist. Lahore, Punjab, Pakistan, and were authenticated by the Herbarium Department of Botany, Lahore College for Women University, Lahore. Rhizomes were dried in an oven at 50°C–60°C for 24 h. For the preparation of the aqueous extract, the dried ginger rhizome was ground. One hundred grams of dried ginger powder was added to 3000 mL of distilled water (H_2_O) under constant stirring at 100 rpm for 2 h. The solution was filtered, and the filtrate was allowed to evaporate under vacuum. The crude extract was preserved for further analysis [10].

EO extraction was done using 4000 g of ginger rhizome added in 400 mL of distilled H_2_O in a 1000 mL round‐bottomed flask. EO was extracted using a Clevenger‐type apparatus through hydrodistillation methods. The crude oil was collected into a separating funnel, where it was separated from the upper layer. For further analysis, the EO was then dried over anhydrous sodium sulfate at −4°C. The dilutions of both extracts (aqueous extract and EO) were prepared in DMSO ranging from 200, 100, 50, 25, 12.5, 6.25, and 3.125 mg mL^−1^ by following the methodology of [11].

### 2.2. Antioxidant Estimation

The antioxidant profile of both extracts (aqueous and EO) of ginger rhizome was evaluated through the 2,2‐diphenyl‐1‐picrylhydrazyl (DPPH) free radical scavenging assay, total phenolic content (TPC), and total antioxidant assays (TAAs).

### 2.3. DPPH Free Radical Scavenging Assay

Free radical scavenging (percentage) of both ginger extracts (aqueous extract and EO) was determined by the DPPH assay. To perform this, 1 × 10^−4^ M DPPH was added in 100 mL of DMSO in amber glass jars kept for 30 min. One milliliter of plant extract was added in 1 mL of DPPH solution and adjusted the volume up to 4 mL by DMSO [11]. The solution was incubated for 30 min. The UV visible spectrophotometer (GIORGIO‐BORMAC SRL, Carpi, Italy) was used to measure the absorbance at 517 nm. The blank was prepared with the DMSO dilution of DPPH. The results were expressed in milligram equivalents of alpha‐tocopherol per milligram of dry weight. The formula used to compute DPPH inhibition was

%DPPH=1−Abs sampleAbs control×100.

### 2.4. TPC Assay

TPC of both extracts was determined by following the methodology of Aftab et al. [12]. Then, 0.4 mL of both extracts at the entire range of concentrations was added in 2 mL of 2 N Folin–Ciocalteu reagent and 1.6 mL of 10% sodium carbonate (Na_2_CO_3_). The absorbance was recorded at 725 nm by using a UV‐visible spectrophotometer. TPC was estimated as micrograms per gram of dry weight using a gallic acid equivalent standard calibration curve created through *y* = 0.005*x* + 0.047 while *R* ^2^ = 0.998 using various concentrations of gallic acid.

### 2.5. TAA

The total antioxidant content was determined by phosphomolybdenum reagent solution, prepared by mixing the solution of 28 mM sodium phosphate, 0.6 M sulfuric acid, and 4 mM ammonium molybdate. The EO from each concentration and phosphomolybdenum reagent solution was added in equal concentrations in each test tube. The reaction mixture was allowed to cool at room temperature after 60 min at 95°C of incubation. The absorbance was measured at 695 nm [11].

### 2.6. Antimicrobial Activity

Antimicrobial activity was checked through the agar well diffusion method by following the methodology of Naeem et al. [13]. Three bacterial strains (*Azospirillum lipoferum* FCBP‐PB‐0434, *Azospirillum brasilense* FCBP‐SB‐0025, and *Pantoea agglomerans* FCBP‐PB‐0454) and three fungal strains (*Trichoderma hamatum* FCBP‐PTF‐769, *Trichoderma harzianum* FCBP‐SF‐1277, and *Trichoderma viride* FCBP‐DNA‐639) were used.

For culturing microbial strains, 18 g of malt extract and 15 g of agar were added to 10 mL of distilled H_2_O under constant stirring, and then the volume was raised to 1000 mL. The solution was homogenized and autoclaved (121°C) at 15 Ib^2^/inch pressure. Twenty milliliters of media was poured into each petri plate; the solution was solidified at room temperature. The prepared plates were inoculated with microbial strains. A cork borer was used to make the wells, and the sample was poured into the wells. The petri plates were incubated at 37°C for 24 h (bacterial strains) and at 30°C for 5–7 days (fungal strains), and the zone of inhibition (millimeters) was measured.

### 2.7. Antidiabetic Activity

Antidiabetic activity was done through *α*‐amylase inhibition assay by following the methodology of Saeed et al. [14]. Alpha‐amylase (0.5 mg mL^−1^) was mixed in 500 *μ*L of 0.02 M sodium phosphate buffer (adjusting its pH to 6.9 with 0.006 M sodium chloride) and incubated for 10 min at 25°C. Two hundred microliters of both extracts (EO and aqueous) were added to 500 *μ*L of a 1% starch solution (prepared in 0.02 M PBS). Then, 1.0 mL of dinitrosalicylic (DNS) acid was added to stop the reaction. The reaction mixture was kept in a boiling H_2_O bath for 5 min, then allowed to cool at room temperature. The reaction mixture was diluted by adding 5 mL of distilled H_2_O. The absorbance was determined by using a UV‐visible spectrophotometer at the wavelength of 540 nm. The absorbance measurements were compared with *α*‐amylase as the standard control. The formula used to determine the percentage of *α*‐amylase inhibition was

%α−amylase inhibition=Abs of Control–Abs of SampleAbs of Control×100.

### 2.8. Anti‐Inflammatory Activity

Anti‐inflammatory activity was done by following the methodology of Dinesh et al. [15]. Then, 0.1 mL of both extracts of ginger rhizome was added to 0.9 mL of bovine serum albumin. The prepared reaction mixture was incubated for 20 min at 37°C. After the incubation, the reaction mixture was kept in a boiling H_2_O bath for 30 min at 55°C. Then, 2.5 mL of phosphate buffer (pH 6.3) was added to the reaction mixture and then allowed to cool at room temperature. The absorbance was measured at 660 nm. Diclofenac sodium was used as the standard control. The formula used to calculate the percentage of protein denaturation was

%Inhibition=Abs of Control–Abs of SampleAbs of Control×100.

### 2.9. FTIR Spectroscopy

FTIR spectroscopy was performed to determine the functional groups present in both extracts using an FTIR spectrometer at a resolution of 4 cm^−1^. Ten milligrams of the dried extract powder was encapsulated in 100 mg of KBr pellet in order to prepare translucent sample discs. The powdered sample was loaded in the FTIR spectroscope (Shimadzu, IR Affinity 1, Japan) with a scan range from 400 to 4000 cm^−1^ range. The room′s relative humidity was maintained at 30% [16].

### 2.10. GCMS Analysis

GCMS of ginger rhizome aqueous and EO extracts was done by following the methodology of Tunnisa et al. [17]. GC (Shimadzu Nexis GC‐2030) was coupled to the MS detector (Shimadzu GC‐MS‐QP2020 NX). At analysis conditions, a stabilized wax column (60 m, 0.25 mm ID, film thickness of 0.25 m), an injector temperature of 80°C, and temperature control for the column are raised in a gradient by 4°C/min until it reaches 150°C after being kept at 40°C for 5 min. The temperature was then raised by 30°C/min until it reached its final setting of 250°, which was held for 5 min. In split injection mode, helium was employed as the mobile phase at 1 mL min^−1^. The MS condition was run in EI ionization mode; the detector and interface temperatures were 230°C and 250°C, respectively. It ran for 40 min. By contrasting the mass spectra with the NIST Library (NIST14 standard version) and the linear retention index (LRI) value with outside standards, compounds were identified. The LRIs of each component were calculated using an identical series of an n‐alkane solution (C10‐40, Polyscience, Niles, IL, United States; 5 mg L^−1^) in dichloromethane under the same chromatographic parameters as the samples.

### 2.11. LCMS Analysis

EO extract with the best pharmacological potential was subjected to LCMS analysis for the identification of bioactive compounds. One milliliter of 10 ppm aqueous fraction was prepared, and 10 *μ*L was auto‐injected into the LCMS with a solvent system of 95% ACN (acetonitrile): 5% H_2_O through a Poroshell 120 EC‐C_18_ column (4.6 × 150 mm, 4 *μ*m) with a column temperature of 35°C and a flow rate of 0.5 mL/min. Full total ion chromatogram (TIC) scans in positive mode were obtained for ion identification. All analytes were evaded in 31.902 min [12]. Compounds with their chemical structure and fragments were identified by comparing their retention time (RT) and mass spectra to those of the WILEY 09, NIST 11, ChemSpider, and muse mass spectral databases Version 12.0 [18]. The mean and standard deviation were used to calculate the data, and a two‐way analysis of variance was performed using SPSS Version 22. For correlation, Origin 2016 with Python was employed.

## 3. Results and Discussion

The observations from various activities were later analyzed through ANOVA analysis using SPSS, which indicated a significant relationship among all activities, with a significance level of *p* < 0.05. Correlation analysis showed the significant relationship between all pharmacological activities.

### 3.1. Antioxidant Activities

Ginger exhibits antioxidant potential because of the presence of polyphenol content and hence enhances the functionality of foods [19]. In the present study, TPC, DPPH radical scavenging percentage, and TAA determined the antioxidant potential of EO and aqueous extract of ginger rhizome. Results showed that ginger rhizome was observed to have significant (*p* < 0.05) antioxidant potential (Figure 1). An increase in concentration was observed to cause an increase in antioxidant activity. Aqueous extract was observed to have maximum TPC as compared to EO among all concentrations. Maximum TPC (399 ± 1^g^ mg GAE/g) was observed in aqueous extract, followed by (270 ± 1^g^ mg GAE/g) in EO at 200 mg mL^−1^ concentrations. The DPPH free radical scavenging values (percentage) of both extracts of ginger rhizomes ranged from 60.93^g^ %. Aqueous extracts had significantly higher TAA (1.95 ± 0.05^g^) at 200 mg mL^−1^ followed by 1.82 ± 0.05^f^ at 100 mg mL^−1^ concentration. Antioxidants are substances that protect cells from oxidative damage caused by free radicals. In this study, various assays were conducted to measure the antioxidant activity of *Z. officinale* Roscoe, including TPC assay, DPPH free radical scavenging assay, and TAA. Plant‐based antioxidant molecules provide electrons and hydrogen ions to prevent cellular damage. The antioxidant capacity of EOs was evaluated using the DPPH assay [20], where the change in color from violet to yellow indicated antioxidant activity. The level of antioxidant activity was found to be concentration‐dependent, with higher concentrations showing stronger antioxidant activity [21]. In this study, the Folin–Ciocalteu reagent was used to measure the TPC. The EO of *Z. officinale* exhibited a maximum TPC of 270 ± 1 at 200 mg mL^−1^, while the aqueous extract showed a maximum TPC of 399 ± 1 mg mL^−1^ at the same concentration. The study by Kamaliroosta et al. [22] focused on the TPC assay of *Z. officinale*, identifying EO components. Phenolic compounds in *Z. officinale* are known for their redox characteristics, singlet oxygen quenching, and hydrogen donation properties.

Figure 1Antioxidant activity of different extracts of ginger rhizome. (a) Total phenolic content, (b) DPPH free radical scavenging activity, and (c) total antioxidant assay.

**Figure:**
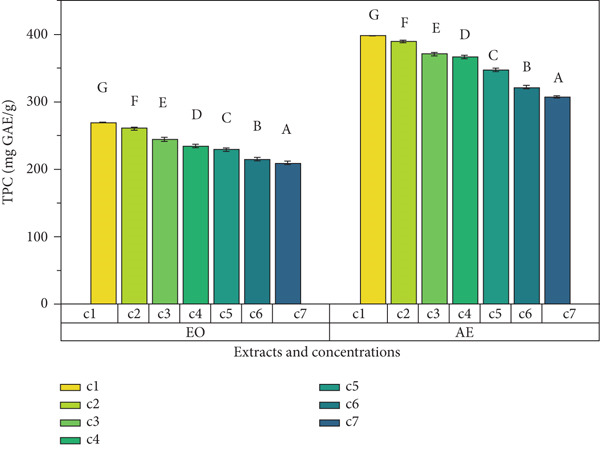
(a)

**Figure:**
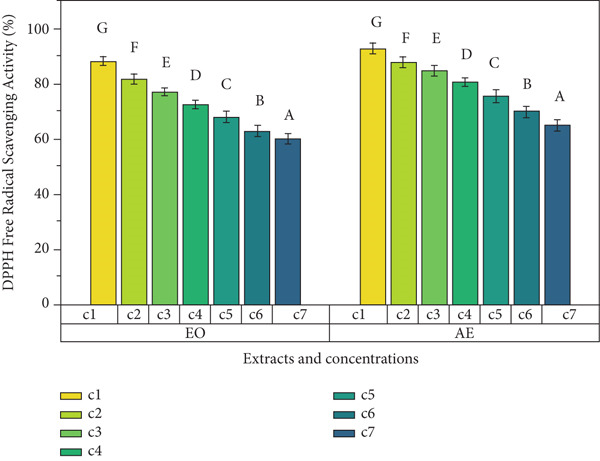
(b)

**Figure:**
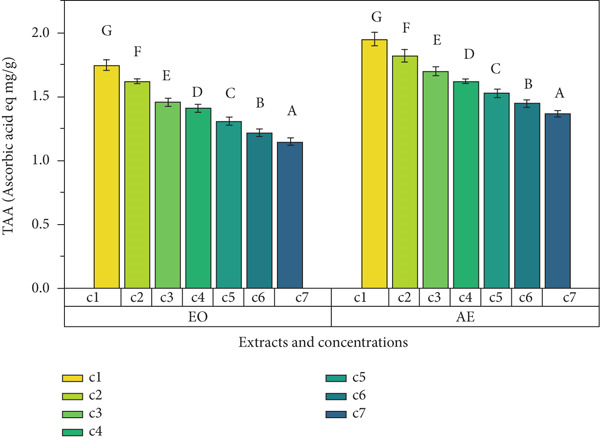
(c)

DPPH assay was used to determine the radical scavenging activity of the samples. The EO of *Z. officinale* exhibited a maximum DPPH radical scavenging activity of 60.93% at 200 mg mL^−1^, while the aqueous extract showed 93*%* ± 2*%* at the same concentration. Ismaeel and Usman [23] confirmed that the antioxidant activity of *Z. officinale* is attributed to its hydrogen donation capacity, with activity increasing with concentration.

TAA measured the absorbance of the samples, with the EO of *Z. officinale* showing a maximum antioxidant activity of 1.75 ± 0.04 μg/g of gallic acid at 200 mg mL^−1^ and the aqueous extract showing 1.95 ± 0.05 μg/g of gallic acid at the same concentration. Thinh and Thin [24] reported that the presence of 2,5‐dimethoxy‐p‐cymene in the EO contributes to its high antioxidant activity.

### 3.2. Antimicrobial Activity

Ginger showed potent antimicrobial potential against the tested microbial strains. All the tested microbial strains showed sensitivity against both extracts of ginger rhizome. The results showed that the antimicrobial potential of ginger rhizome is dose dependent (Table 1). Minimum zones of inhibition (millimeters) against tested strains were observed by DMSO (negative control). Among fungal strains, the EO extract showed the maximum zone of inhibition (millimeters), that is, 31.66 ± 1.52^g^ and 21.33 ± 2.51^f^ against *T. hamatum* and *T. viride*, respectively, at 200 mg mL^−1^, whereas the aqueous extract showed the highest antifungal potential against *T. harzianum* (25.66 ± 2.51^f^). Bacterial strains tested also showed sensitivity against both extracts at the entire range of concentrations. The aqueous extract was observed to have the highest inhibition activity against *A. lipoferum*, that is, 28 ± 3^f^ at 200 mg mL^−1^ concentration, whereas at 200 mg mL^−1^, EO showed maximum inhibitory zones against *A. brasilense* (26 ± 2^e^) and *P. agglomerans* (21 ± 2^d^), respectively. Antimicrobial activity was assessed by measuring the zone of inhibition. The EO and aqueous extract of *Z. officinale* exhibited antibacterial activity against various strains, with the EO showing maximum inhibition against lipoferum. Das et al. [25] highlighted the presence of phytochemicals like *α*‐zingiberene, *α*‐curcumene, and terpenes in *Z. officinale*, contributing to its antimicrobial potential. The antifungal potential of *Z. officinale* was demonstrated by its ability to inhibit the growth of *T. hamatum*, with the EO and aqueous extract showing significant antifungal activity. Akarchariya et al. [26] and Mushtaq et al. [27] attributed this activity to the presence of compounds like p‐cymen‐7‐ol and terpinolene.

**Table 1:** Antimicrobial activity of different extracts of ginger rhizome against various microbial strains.

**Conc. (mg mL** ^**−1**^ **)**	**Essential oil**	**Aqueous extract**	**Essential oil**	**Aqueous extract**	**Essential oil**	**Aqueous extract**
Fungus	*Trichoderma harzianum*		*Trichoderma hamatum*		*Trichoderma viride*	
200	24 ± 2^f^	25.6 ± 2.51^f^	31.66 ± 1.52^g^	27.66 ± 2.51^g^	21.33 ± 2.51^f^	20.33 ± 1.52^f^
100	22 ± 2.1^ef^	20 ± 2^ef^	27.66 ± 2.51^fg^	25.33 ± 2.51^fg^	20.33 ± 1.5^ef^	17.6 ± 2.5^ef^
50	20 ± 2.5^de^	15.33 ± 2.51^cd^	25 ± 2^ef^	21.33 ± 2.51^ef^	17.6 ± 1.5^def^	17 ± 2^def^
25	18 ± 1.5^cd^	17 ± 2^de^	23 ± 2^de^	20 ± 2^de^	15.3 ± 2.5^bcde^	15.3 ± 2.5^bcde^
12.5	14 ± 2^bc^	13 ± 2^bc^	20 ± 2^cd^	17.66 ± 2.51^cd^	14.3 ± 2.51^bcd^	14 ± 2^bcd^
6.25	12 ± 2^b^	12.3 ± 1.52^b^	17.66 ± 2.51^bc^	16 ± 2^bc^	13 ± 2^bc^	12.66 ± 1.52^bc^
3.125	11 ± 2^b^	11.33 ± 1.52^b^	15 ± 2^b^	14 ± 2^b^	11.66 ± 1.52^b^	12 ± 1^b^
Fluconazole	18 ± 3^de^	18 ± 3^de^	24.33 ± 1.52^ef^	24.33 ± 1.52^ef^	16.33 ± 1.5^cde^	16.33 ± 1.52^cde^
DMSO	9.3 ± 1.5^a^	9.33 ± 1.52^a^	10.33 ± 1.52^a^	10.33 ± 1.52^a^	8.33 ± 1.52^a^	8.33 ± 1.52^a^
Bacteria	*Azospirillum lipoferum*		*Azospirillum brasilense*		*Pantoea agglomerans*	
200	27.66 ± 2.51^f^	28 ± 3^f^	26 ± 2^e^	24 ± 2^e^	21 ± 2^d^	18 ± 2^d^
100	23.33 ± 2.51^ef^	24.66 ± 2.51^ef^	19.33 ± 2.51^cde^	22.66 ± 2.51^cde^	18 ± 2^cd^	15.33 ± 2.51^cd^
50	20 ± 2^de^	20 ± 2^de^	18 ± 3^bcd^	18.66 ± 2.51^bcd^	14.66 ± 2.51^bc^	14.66 ± 2.51^bc^
25	17.33 ± 2.51^cd^	18.66 ± 2.51^cd^	16.66 ± 2.51^bc^	17.33 ± 2.51^bc^	14 ± 2^bc^	14 ± 2^bc^
12.5	15.33 ± 2.51^bc^	15 ± 2^bc^	15.33 ± 2.51^b^	16.66 ± 2.08^b^	13 ± 2^b^	12.33 ± 2.51^b^
6.25	14 ± 3^ab^	12.33 ± 2.51^ab^	14.33 ± 2.51^b^	15.66 ± 2.08^b^	12 ± 2^b^	11.66 ± 1.52^b^
3.125	12.33 ± 2.51^ab^	11 ± 2^ab^	13.33 ± 2.51^b^	14.66 ± 2.08^b^	10.66 ± 2.08^b^	11 ± 1^b^
Amoxicillin	23.66 ± 3.05^ef^	23.66 ± 3.05^ef^	22.33 ± 2.51^de^	22.33 ± 2.51^de^	17.33 ± 2.51^cd^	17.33 ± 2.51^cd^
DMSO	9.66 ± 1.52^a^	9.66 ± 1.52^a^	9 ± 1^a^	9 ± 1^a^	8.33 ± 1.52	8.33 ± 1.52^a^

*Note:* Different superscript letters indicate data significance and range.

### 3.3. Antidiabetic Activity

The antidiabetic activity of ginger rhizome extracts was determined using alpha amylase inhibitory activity, and acarbose was used as a standard. The present study showed that ginger rhizome EO exhibits the highest alpha amylase inhibitory potential (67.33 ± 1.52^g^) followed by aqueous extract (59.53 ± 1.85^g^) at 200 mg mL^−1^ (Table 2). In terms of antidiabetic potential, the EO of *Z. officinale* exhibited higher activity compared to the aqueous extract, with compounds like 6‐gingerol contributing to its antidiabetic effects. Dhanik et al. [28] reported that these compounds enhance insulin production [29] and protect beta cells. Acarbose is a complex oligosaccharide that acts as a competitive, reversible inhibitor of pancreatic alpha‐amylase and membrane‐bound intestinal alpha‐glucoside hydrolase. Pancreatic alpha‐amylase hydrolyzes complex carbohydrates to oligosaccharides in the small intestine. Intestinal alpha‐glucosidase hydrolase breaks down oligosaccharides, trisaccharides, and disaccharides (sucrose and maltose) to monosaccharides (glucose and fructose) in the brush border of the small intestine. By delaying the digestion of carbohydrates, acarbose slows glucose absorption, reducing postprandial glucose blood concentrations [30].

**Table 2:** Antidiabetic and anti‐inflammatory activities of ginger rhizome extracts.

**Concentrations (mg mL** ^**−1**^ **)**	**Antidiabetic**		**Anti-inflammatory**	
	**Essential oil**	**Aqueous extract**	**Essential oil**	**Aqueous extract**
200	67.33 ± 1.52^g^	59.53 ± 1.85^g^	83.33 ± 0.90^g^	87.32 ± 0.82^g^
100	40.8 ± 1.70^e^	40.36 ± 1.58^e^	72.23 ± 0.93^f^	79.46 ± 0.84^f^
50	35.76 ± 1.66^d^	23.86 ± 1.80^d^	71.65 ± 0.78^d^	66.83 ± 0.93^d^
25	26.73 ± 1.61^cd^	21.76 ± 1.66^cd^	70.78 ± 0.96^cd^	65.87 ± 0.99^cd^
12.5	23.9 ± 1.85^bc^	19.43 ± 1.69^bc^	69.92 ± 0.95^bc^	64.62 ± 0.75^bc^
6.25	21.96 ± 1.95^b^	17.5 ± 1.80^b^	68.80 ± 0.89^b^	63.54 ± 0.64^b^
3.125	18.76 ± 1.66^a^	15.96 ± 1.95^a^	60.68 ± 0.78^a^	58.81 ± 0.99^a^
Standard	56.7 ± 1.57^f^	56.7 ± 1.57^f^	73.56 ± 0.67^e^	73.56 ± 0.67^e^

*Note:* Different superscript letters indicate data significance and range.

### 3.4. Anti‐Inflammatory Activity

For the determination of anti‐inflammatory activity, diclofenac sodium (200 mg mL^−1^) was used as a positive control. Maximum anti‐inflammatory activity was determined for the ginger rhizome aqueous extract (87.32 ± 0.82^g^) followed by EO extract (83.33 ± 0.90^g^) obtained by hydrodistillation method at 200 mg mL^−1^ concentration (Table 2). The anti‐inflammatory activity of *Z. officinale* was demonstrated by its ability to reduce inflammation, with compounds like gingerol and camphene playing a key role. Ezzat et al. [29] highlighted the anti‐inflammatory properties of *Z. officinale*, attributing it to the inhibition of inflammatory pathways.

**Figure 2:**
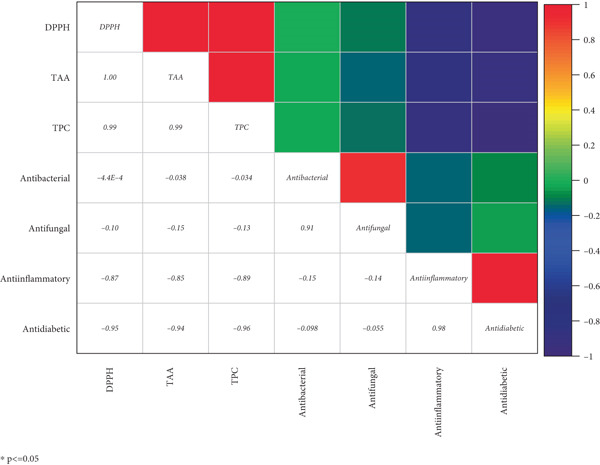
Correlation coefficients of total phenolic contents, DPPH, total antioxidant assay, and biological activities from ginger rhizome extracts using Pearson′s correlation. Different *R* values of Pearson′s correlations are illustrated by various square colors.

The correlation (Pearson) between TPC, DPPH, TAA, and biological activities (antimicrobial, anti‐inflammatory, and antidiabetic activity) is analyzed and presented in Figure 2. The results showed both positive and negative correlations. The correlation was *r* ^2^ = 0.97 between DPPH (free radical scavenging percentage) and TAA. DPPH was also observed to have a strong correlation with TPC. Furthermore, a significant negative correlation was observed between alpha‐amylase inhibitory assay, DPPH, and TPC, that is, *r* ^2^ = −0.219 and −0.32, respectively, whereas alpha‐amylase inhibitory activity was observed to have a moderate positive correlation with anti‐inflammatory, antifungal, and antibacterial activities at *r* ^2^ = 0.8, 0.28, and 0.2, respectively.

### 3.5. FTIR Spectroscopy

Various peaks shown in Figure 3a,b revealed that fractions of both ginger rhizome extracts contained complex molecules. The EO extract has different functional groups at different peaks such as at 3737 cm^−1^ (hydroxyl group), 2928 cm^−1^ (hydrocarbons), 1677 cm^−1^ (carbonyl group), 1358 cm^−1^ (carboxyl group), and 602 cm^−1^ (hydrocarbon group). Aqueous extract fraction showed different peaks at 3751, 2926, 1689, 1370, and 638 cm^−1^ exposed OH, CH group of saturated hydrocarbons, C=O, C–O group, and CH group, respectively (Table 3). FTIR analysis was used to depict the EO and aqueous extract, revealing different functional groups such as alkenes, primary amines, and alcohols. Peaks at 3737, 2928, 1677, and 1358 cm^−1^indicated various functional groups. The study by Varghese et al. [31] described the FTIR of *Z. officinale*, showing peaks at 3447, 2078, 1636, and 547 cm^−1^.

Figure 3FTIR Spectra of ginger rhizome. (a) Essential oil fraction and (b) aqueous fraction.

**Figure:**
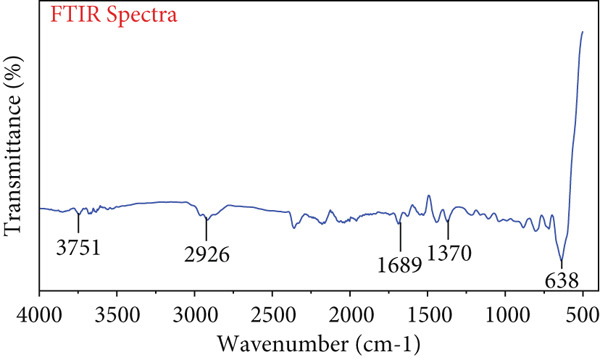
(a)

**Figure:**
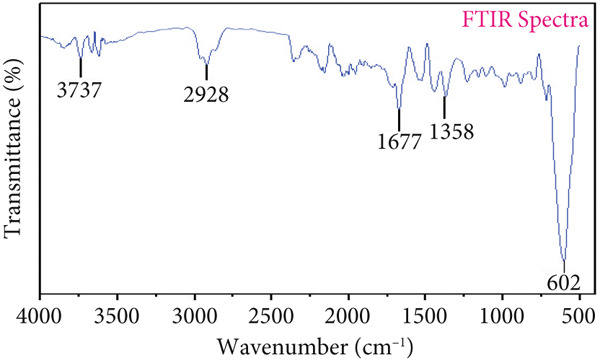
(b)

**Table 3:** FTIR analysis of essential oil and aqueous extract fraction of ginger rhizome.

**Extracts**	**Peaks**	**Functional groups**
Essential oil	3737	OH group
	2928	CH group of saturated hydrocarbons
	1677	C=O group
	1358	C–O group
	602	CH group

Aqueous extract	3751	OH group
	2926	CH group of saturated hydrocarbons
	1689	C=O group
	1370	C–O group

### 3.6. GCMS Analysis

GCMS of EO and aqueous extract fraction of ginger rhizome reveals 23 compounds in aqueous extract (Figure 4a,b) and 32 compounds in EO extract (Tables 4 and 5). Both extracts are observed to have strong potential antioxidant, antimicrobial, alpha‐amylase inhibitory, and anti‐inflammatory potential due to the presence of different phytochemicals like phenolic acids (OH group), flavonoids (C=O, carbonyl group), gingerols (OH group), and derivatives [32]. GCMS analysis identified major compounds in *Z. officinale*, including camphene, methyl heptenone, 4‐carene, terpinolene, citronellal, borneol, linalool, nerol, alpha‐terpineol, alpha‐zingiberene, gamma cadinene, and epiglobulol. Another study by Xu et al. ([33, 34]) reported the presence of calamenen, shogaol, sabinene, gingerol, alpha‐muurolene, paradol, alpha‐cadinol, carvenone, acoradiene, curzerene, beta‐eudesmol, cubenol, beta‐santalol, longiborneol, and aromadendrene, in which shogaol and zingiberol were also determined from the present investigation. Likewise, the presence of shogaol, geraniol, and linalool was confirmed by Amriati et al. [35]. Another article reported by [36] confirmed the presence of dihydroxybenzoic acid, tetramethylsilyl, and tetradecamethyl heptasiloxane, which were found to be different from the present study.

Figure 4Radar chart displaying GC‐MS analysis of ginger rhizome. (a) Aqueous extract and (b) essential oil.

**Figure:**
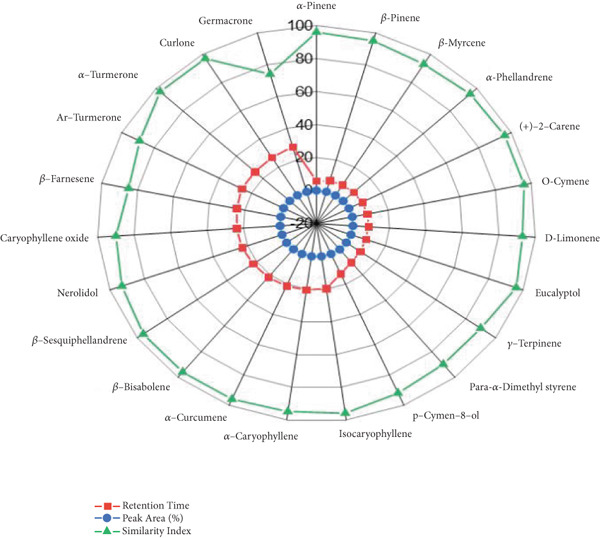
(a)

**Figure:**
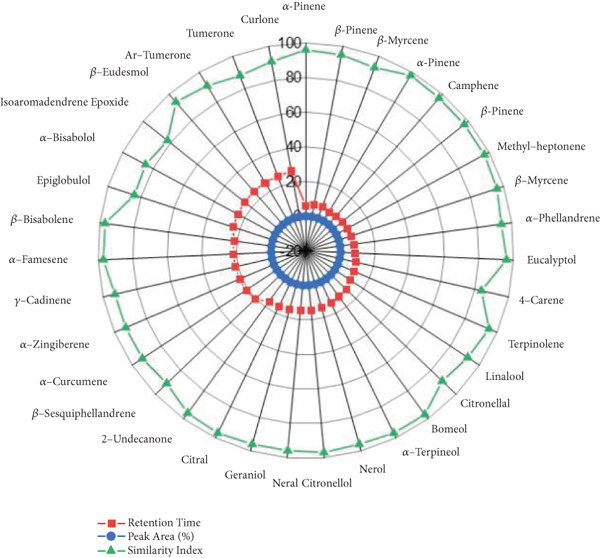
(b)

**Table 4:** GCMS analysis of aqueous extract fraction of ginger rhizome.

**Sr. no.**	**Compound name**	**Mol. formula**	**Structure**	**Retention time**	**Peak area (%)**	**Similarity index**	**Mol. mass**
1	Alpha‐pinene	C_10_H_16_		5.875	0.25%	96	136

2	Beta‐pinene	C_10_H_16_		7.017	0.14%	95	136

3	Beta‐myrcene	C_10_H_16_		7.358	0.14%	93	136

4	Alpha‐phellandrene	C_10_H_16_		7.792	0.36%	95	136SSS

5	(+)‐2‐Carene	C_10_H_16_		8.075	1.65%	96	136

6	O‐Cymene	C_10_H_14_		8.325	1.85%	96	134

7	D‐Limonene	C_10_H_16_		8.442	0.86%	93	136

8	Eucalyptol	C_10_H_18_O		8.517	4.40%	96	154

9	Gamma‐terpinene	C_10_H_16_		9.317	0.076%	90	136

10	Para‐alpha‐dimethyl styrene	C_10_H_12_		10.267	18.77%	90	132

11	p‐Cymen‐8‐ol	C_10_H_14_O		13.167	0.91%	92	150

12	Isocaryophyllene	C_15_H_24_		19.675	7.48%	96	204

13	Alpha‐caryophyllene	C_15_H_24_		20.608	3.07%	95	204

14	Alpha‐curcumene	C_15_H_22_		21.275	2.50%	96	202

15	Beta‐bisabolene	C_15_H_24_		21.950	0.74%	96	204

16	Beta‐sesquiphellandrene	C_15_H_24_		22.392	4.58%	96	204

17	Nerolidol	C_15_H_26_O		23.317	0.77%	93	222

18	Caryophyllene oxide	C_15_H_24_O		23.858	0.59%	90	220

19	Beta‐farnesene	C_15_H_24_		24.650	0.28%	85	204

20	Ar‐turmerone	C_15_H_20_O		26.017	24.01%	89	216

21	Alpha‐turmerone	C_15_H_22_O		26.108	12.32%	97	218

22	Curlone	C_15_H_22_O		26.808	7.18%	97	218

23	Germacrone	C_15_H_22_O		28.408	0.23%	74	218

**Table 5:** GCMS analysis of essential oil extract of ginger rhizome.

**Sr. no.**	**Compound name**	**Molecular formula**	**Structure**	**Retention time**	**Peak area (%)**	**Similarity index**	**Molecular mass**
1	Alpha‐pinene	C_10_H_16_		5.883	3.24%	98	136

2	Camphene	C_10_H_16_		6.325	9.10%	97	136

3	Beta‐p	C_10_H_16_		7.008	0.53%	97	136

4	Methyl heptenone	C_8_H_14_O		7.258	1.33%	97	126

5	Beta‐myrcene	C_10_H_16_		7.325	2.78%	96	136

6	Alpha‐phellandrene	C_10_H_16_		7.783	0.43%	94	136

7	Eucalyptol	C_10_H_18_O		8.567	15.18%	96	154

8	4‐Carene	C_10_H_16_		9.292	1.65%	84	136

9	Terpinolene	C_10_H_16_		10.050	0.33%	95	136

10	Linalool	C_10_H_18_O		10.517	1.15%	92	154

11	Citronellal	C_10_H_18_O		12.017	0.35%	89	154

12	Borneol	C_10_H_18_O		12.675	1.58%	97	154

13	Alpha‐terpineol	C_10_H_18_O		13.383	1.46%	97	154

14	Nerol	C_10_H_18_O		14.267	0.97%	96	154

15	Citronellol	C_10_H_20_O		14.358	1.96%	97	156

16	Neral	C_10_H_16_O		14.742	13.50%	96	152

17	Geraniol	C_10_H_18_O		15.067	3.84%	96	154

18	Citral	C_10_H_16_O		15.633	17.32%	97	152

19	2‐Undecanone	C_11_H_22_O		16.208	0.42%	96	170

20	Beta‐sesquiphellandrene	C_15_H_24_		20.542	1.81%	91	204

21	Alpha‐curcumene	C_15_H_22_		21.283	2.81%	93	202

22	Alpha‐zingiberene	C_15_H_24_		21.683	8.98%	93	204

23	Gamma‐cadinene	C_15_H_24_		21.750	1.16%	93	204
24	Alpha‐farnesene	C_15_H_24_		21.892	4.11%	97	204

25	Beta‐bisabolene	C_15_H_24_		21.975	1.80%	97	204

26	Epiglobulol	C_15_H_26_O		24.067	0.36%	84	222

27	Alpha‐bisabolol	C_15_H_26_O		24.642	0.25%	85	222

28	Isoaromadendrene epoxide	C_15_H_24_O		25.058	0.32%	82	220

29	Beta‐eudesmol	C_15_H_26_O		25.650	0.35%	94	222

30	Ar‐turmerone	C_15_H_20_O		25.850	0.49%	91	216

31	Tumerone	C_15_H_22_O		25.967	0.34%	88	218
32	Curlone	C_15_H_22_O		26.758	1.61%	91	218

### 3.7. LCMS Analysis

LCMS analysis of the aqueous extract fraction of ginger rhizome was conducted. The LCMS analysis displayed the RT of the possible compounds (Figures 5a, 5b, 5c, 5d, 5e, and 5f). The fragmentation pattern determines the relative abundance of the identified compounds. The compounds having hydroxyl, carbonyl, and hydrocarbon functional groups were also identified through FTIR analysis. LCMS analysis identified compounds such as trihexyl(tetradecyl)phosphonium bis[(trifluoromethyl)sulfonyl]imide, heptatetracontylcyclohexane, 3,5‐dihydroxybenzyl alcohol, tris(heptafluorobutyrate), tetratriacontane, 17‐hexadecyl‐, and 1‐deoxy‐1‐(methylamino)‐D‐galactitol, N,O,O,O,O,O‐hexa(trifluoroacetyl) (Table 6). Tohma et al. [37] testified to the presence of pyrogallol, ferulic acid, vanillin, quercetin, alpha‐tocopherol, and catechol in their LCMS analysis. In another study reported by Tao et al. [38], the presence of 6‐gingerol, 8‐shogoal, 6‐paradol, and 8‐gingerdione in ginger was found to be different from the present investigation. Ashraf et al. [39] reported the presence of 6‐gingerol, 8‐gingerol, and 10‐gingerol.

Figure 5(a–f) LCMS chromatograms of aqueous extract fraction of ginger rhizome.

**Figure:**
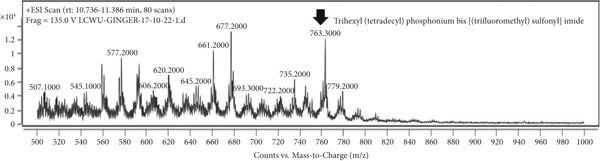
(a)

**Figure:**
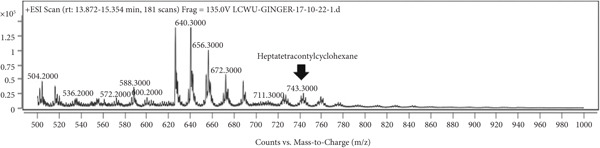
(b)

**Figure:**
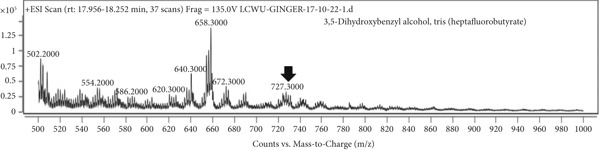
(c)

**Figure:**
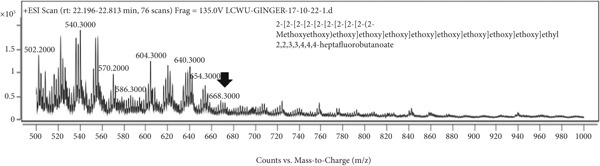
(d)

**Figure:**
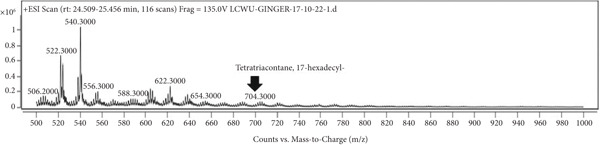
(e)

**Figure:**
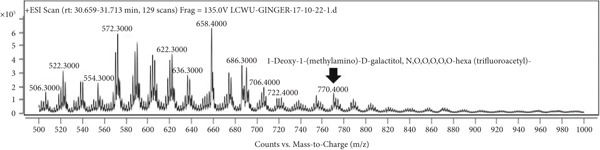
(f)

**Table 6:** Compounds identified by LCMS analysis of aqueous fraction of ginger rhizome.

**Compound**	**Molecular weight (m/z)**	**Molecular formula**	**Base peak (m/z)**	**% area**	**Retention time**	**IUPAC standard InChIKey**	**PubChem ID**
Trihexyl(tetradecyl)phosphonium bis[(trifluoromethyl)sulfonyl]imide	764.002	C_34_H_68_F_6_NO_4_PS_2_	559	3.3	11.5–12.6	HYNYWFRJHNNLJA‐UHFFFAOYSA‐N	11181836
Heptatetracontylcyclohexane	743.4	C_53_H_106_	640	13.7	13.8–15.3	MFNVIUZFJNHENR‐UHFFFAOYSA‐N	129658656
3,5‐Dihydroxybenzyl alcohol, tris(heptafluorobutyrate)	728.206	C_19_H_5_F_21_O_6_	658	4.3	17.9–18.2	LEMOQPWTCMZOIO‐UHFFFAOYSA‐N	91725402
Tetratriacontane, 17‐hexadecyl‐	703.3	C_50_H_102_	522	45.85	24.5–25.4	DAQUEPISOMDKQG‐UHFFFAOYSA‐N	
2‐[2‐[2‐[2‐[2‐[2‐[2‐[2‐[2‐(2‐Methoxyethoxy)ethoxy]ethoxy]ethoxy]ethoxy]ethoxy]ethoxy]ethoxy]ethoxy]ethyl 2,2,3,3,4,4,4‐heptafluorobutanoate	668.6	C_25_H_43_F_7_O_12_	502	17.86	30.6–31.7	VPJYVICSTNIRTJ‐UHFFFAOYSA‐N	
1‐Deoxy‐1‐(methylamino)‐D‐galactitol, N,O,O,O,O,O‐hexa(trifluoroacetyl)‐	771.26	C_19_H_11_F_18_NO_11_	572	74.4	30.6–31.7	AINUEIDIXPMVIA‐UHFFFAOYSA‐N	91716731

Table 4 showed the identified compounds in the aqueous extract fraction of ginger rhizome. The functional groups identified through FTIR and GCMS/LCMS showed close association. The compounds identified through LCMS mostly had OH and CH functional groups, whereas the compounds identified through GCMS had all CH, C=O, and OH functional groups.

Medicinal plants play a crucial role in various industries such as food, perfumery, cosmetics, and pharmaceuticals [40]. The demand for medicinal plants is increasing globally due to their natural properties and minimal side effects compared to synthetic medications. Synthetic drugs have been associated with serious side effects [41], leading to approximately 100,000 deaths annually. As a result, there is a growing trend toward herbal therapies that offer safer alternatives [42]. In this study, the effectiveness of EO and aqueous extract of *Z. officinale* Roscoe was evaluated for various pharmaceutical activities. The EO was extracted using a Clevenger‐style apparatus, as the hydrodistillation technique is known for its ability to extract specific compounds at temperatures below 100°C [43].

## 4. Conclusion

*Z. officinale* Roscoe, a member of the Zingiberaceae family, is a valuable source of natural compounds with wound healing properties. In this study, the EO and aqueous extract of *Z. officinale* were evaluated for their antioxidant, antibacterial, antidiabetic, and anti‐inflammatory activities using various analytical techniques such as GCMS, LCMS, and FTIR spectroscopy.

The aqueous extract of *Z. officinale* exhibited the highest antioxidant potential based on TPC estimation, DPPH assay, and TAA. Both the EO and aqueous extract showed significant inhibition zones against fungal (*T. hamatum*) and bacterial (*A. lipoferum*) strains. Additionally, they demonstrated antidiabetic and anti‐inflammatory properties. The chemical composition of the extracts was analyzed using GCMS and LCMS, while FTIR spectroscopy was used to identify functional groups. The findings of this study suggest that *Z. officinale* extracts have therapeutic benefits with minimal health risks. Incorporating these extracts (aqueous and EO) into pharmaceutical products could offer new and sustainable solutions for various health issues, aligning with Sustainable Goal 3 for health and well‐being.

## Conflicts of Interest

The authors declare no conflicts of interest.

## Author Contributions

Bazghah Sajjad: methodology. Arusa Aftab: research planning and supervision. Zubaida Yousaf: data visualization. Zainab Maqbool: analysis and performance. Wei Sun: data curation. Humaira Rizwana: data visualization and research planning.

## Funding

The authors wish to thank Researchers Supporting Project Number ORF‐2025‐1048 at King Saud University, Riyadh, Saudi Arabia, for financial support.

## Data Availability

The data that support the findings of this study are available from the corresponding authors upon reasonable request.
